# Effects of feeding pelleted complete diets containing palm fronds on growth performance, nutrient digestibility, intraruminal fermentation, rumen histomorphology, carcass traits, and economic profitability in Awassi lambs

**DOI:** 10.3389/fvets.2026.1864007

**Published:** 2026-05-28

**Authors:** Abdulrahman S. Alharthi, Hani H. Al-Baadani, Ahmed A. Alghonaim, Mohammed Sallam, Ibrahim A. Alhidary

**Affiliations:** 1 Department of Animal Production, College of Food and Agriculture Science, King Saud University, Riyadh, Saudi Arabia; 2 Department of Plant Production, College of Food and Agriculture Science, King Saud University, Riyadh, Saudi Arabia

**Keywords:** economically, lambs, palm frond, performance, ruminal fermentation

## Abstract

The global livestock sector is a cornerstone of food security, but it faces significant pressure to find sustainable alternatives to traditional roughage due to rising costs and environmental constraints in arid regions. Therefore, this study investigated the effects of palm fronds on growth performance, nutrient digestibility, ruminal fermentation, rumen histomorphology, carcass and meat characteristics, and economic profitability in Awassi lambs. Sixty-four male lambs (age: 14 weeks; mean initial weight: 31.69 ± 0.12 kg) were randomly assigned to dietary groups (*n* = 16 per group) receiving pelleted complete diets containing 0, 8, 16%, or 24% palm fronds on a dry matter (DM) basis (A, B, C, and D, respectively). Growth performance indices were assessed over the entire 56-day trial period. At the end of the trial, eight lambs per group were evaluated for nutrient digestibility, and eight were slaughtered to assess intraruminal fermentation, rumen histomorphology, carcass traits, and meat quality. An economic analysis was also performed for each inclusion level. The results showed that the 16% palm frond level (group C) resulted in higher final body weight (BW) and average daily gain (ADG) compared to group A (*p* < 0.05). Although daily feed intake (DFI) decreased in groups B and C, the feed conversion ratio (FCR) improved in group C (*p* < 0.05). Nutrient digestibility (DM, CP, NDF, and ADF) peaked at the 16% inclusion level (*p* < 0.05), while ash and fat digestibility were unaffected in all groups. Additionally, 16% palm frond inclusion enhanced rumen development by increasing papillae length, surface area, and total volatile fatty acid (*p* < 0.05). Lambs fed 16% palm fronds had greater carcass weight (hot and cold), springiness, dressing percentage, and shoulder percentage, as well as reduced cold shrink and water-holding capacity (*p* < 0.05). However, palm frond inclusion (16 and 24% levels) supported normal muscle development while favorably redirecting energy from visceral fat deposition toward leaner carcass growth. Economically, while the 24% level minimized feed costs, the 16% level maximized net return and return on investment. In conclusion, incorporating palm fronds at a 16% level provides an optimal balance between physiological adaptation and economic efficiency for sustainable sheep production.

## Highlights

- Lambs fed pelleted complete diets containing 16% palm fronds showed improved performance and nutrient digestibility (DM, CP, NDF and ADF).
- The 16% inclusion level increased volatile fatty acid production and promoted rumen development by increasing papillae length and surface area.
- This substitution supported leaner carcass growth and reduced relative cold shrink in Awassi lambs.
- Economic analysis confirmed that 16% inclusion maximizes net return and provides the highest price safety threshold.

## Introduction

The global livestock industry faces a dual challenge: rising demand for animal protein due to population growth and the simultaneous depletion of natural resources needed for traditional feed production (1). In arid and semi-arid regions, such as the Middle East and North Africa, this challenge is intensified by chronic water scarcity and a heavy reliance on imported feed ingredients, making local food security vulnerable to global market volatility (2). Traditionally, high-quality roughages like alfalfa (*Medicago sativa*) have been the main fiber source for ruminant diets; however, their high-water requirements are increasingly considered ecologically unsustainable in water-stressed environments (3).

Consequently, it is imperative to explore underutilized agricultural by-products that do not compete with human food resources or require additional land or water (4). The date palm (*Phoenix dactylifera L.*) industry is one of the most significant agricultural sectors in the region, with millions of trees producing large quantities of lignocellulosic waste annually (5). A single date palm can produce approximately 13.5–20 kg of fronds and other by-products per year (6, 7). Despite their abundance, these materials are often disposed of by burning or landfilling, leading to environmental degradation and the loss of potentially valuable nutrients (8). Although palm fronds are rich in cellulose and hemicellulose, their use in ruminant nutrition has historically been limited by their high lignin content and low bulk density, which can reduce palatability and alter the rate of passage in the rumen (9). Modern processing through pelleting offers a solution by reducing particle size, increasing dietary density, and preventing selective feeding, thereby enhancing the digestibility of fibrous materials (10).

Previous studies indicate that diets based on palm fronds can improve performance, nutrient digestibility, and economic benefits, without adversely affecting carcass characteristics or meat quality in growing lambs (6, 11). Palm fronds can effectively replace conventional roughage, such as alfalfa or straw, provided inclusion levels are balanced to maintain dietary energy density (7, 12). Soliman et al. (13) reported that lambs fed a pelleted complete diet containing palm fronds showed comparable, and in some cases superior, average daily gains compared to those fed traditional roughage. Furthermore, inclusion of palm fronds can stimulate rumen development and improve the volatile fatty acid profile without compromising the fractional disappearance rate of neutral detergent fiber (14). While higher inclusion levels of palm fronds can reduce feed costs, they may cause a slight plateau in growth due to increased ruminal bulk, which can limit net energy intake (15).

The biological efficacy of these fiber sources is fundamentally linked to rumen development and fermentation biochemistry. Rumen microbiota ferment forage fiber into volatile fatty acids, which provide 50–85% of the primary energy source for ruminants (16). These volatile fatty acids play a multifaceted role in regulating ruminal physiological functions and participate directly in the morphological remodeling of the rumen epithelium by promoting the proliferation and renewal of epithelial cells (17). Recent research highlights that palm frond inclusion stimulates a significant expansion of the rumen’s absorptive surface area by enhancing papilla development (18). This morphological adaptation increases the rate of volatile fatty acid absorption across the ruminal epithelium, supporting the improved feed efficiency observed in lambs fed pelleted complete diet based on palm fronds (19).

Despite previous studies, a comprehensive evaluation of the synergistic effects of graded levels of pelleted palm fronds on the entire production cycle – including growth dynamics, rumen histomorphology, and multidimensional economic profitability – is currently lacking for Awassi lambs. We hypothesized that an optimized inclusion level of palm fronds would enhance growth efficiency and rumen development while significantly improving the economic viability of the production system. Therefore, this study was designed to evaluate the impact of replacing conventional roughage with varying levels of palm fronds in pelleted complete diets on growth performance indices, nutrient digestibility, intraruminal fermentation profiles, rumen histomorphology, carcass and meat characteristics, and economic evaluation in Awassi lambs.

## Materials and methods

### Ethics approve

The Ethics Committee for Research at King Saud University in Riyadh, Saudi Arabia, reviewed and officially approved the experimental protocols and study design. This approval, referenced as KSU-SE-24-73, was granted in accordance with established standards for experimental design and animal welfare.

### Animal management and study design

To evaluate the impact of palm frond inclusion, 64 male Awassi lambs (age: 14 weeks; mean initial weight: 31.69 ± 0.12 kg) were procured from a local source. All lambs were randomly divided using a completely randomized design into 32 pens (4 m^2^) as experimental units, with four groups (*n* = 16 lambs per group) and two lambs per replicate pen. The stocking density of each pen was maintained to facilitate natural movement and social behavior. The experimental diets were formulated as complete pellet diets with different levels of palm fronds at 0, 8, 16, and 24% (groups A, B, C, and D, respectively) to meet NRC (2007) standards for growing lambs. In these formulations, palm fronds served as a partial replacement for the roughage fraction, specifically wheat straw and alfalfa hay. To ensure all diets remained isonitrogenous and isoenergetic, the proportions of the concentrate components, including corn grain, barley grain, corn oil, and soybean meal, were adjusted. The ingredients and nutrient contents for each diet group are shown in Table 1.

**Table 1:** Feed ingredients and nutrient content of dietary groups.

Ingredients (% as-fed)	Palm fronds levels			
	A (0%)	B (8%)	C (16%)	D (24%)
Palm fronds	0.00	8.00	16.00	24.00
Alfalfa hay	21.61	20.93	10.00	0.00
Corn grain	27.52	27.22	27.43	29.10
Barley grain	21.35	21.35	20.42	18.40
Wheat bran	9.12	9.12	7.53	5.45
Soybean meal	10.64	10.68	13.98	17.75
Wheat straw	7.06	0.00	0.00	0.00
Corn oil	1.32	1.32	1.87	2.45
Sodium bicarbonate	0.73	0.73	0.73	0.73
Sodium chloride	0.50	0.50	0.50	0.50
Calcium carbonate	0.00	0.00	1.30	1.30
Diacalcium phosphate	0.00	0.00	0.09	0.17
Premix ^a^	0.15	0.15	0.15	0.15
Total	100	100	100	100
Nutrient analysis (% of DM)				
DM	97.06	95.99	98.93	98.15
OM	91.61	90.27	93.26	92.62
ME, kcal/kg	2,809	2,818	2,815	2,812
CP	15.45	15.52	15.51	15.5
EE	4.63	4.35	4.90	5.47
NDF	31.76	29.61	30.78	32.1
ADF	16.23	15.96	16.9	18.12
Ca	0.61	0.61	0.62	0.61
p	0.39	0.39	0.39	0.39
Ash	5.45	5.72	5.67	5.53

^a^The nutritional profile per kilogram: vitamin A (2,400,000 IU), vitamin D (1,000,000 IU), vitamin E (16,000 IU), and vitamin K (800 mg). Water-soluble vitamins included B1 (600 mg), B2 (1,600 mg), B6 (1,000 mg), B12 (6 mg), biotin (40 mg), folic acid (400 mg), niacin (8,000 mg), and pantothenic acid (3,000 mg). The trace mineral fortification consisted of Co (80 mg), Cu (2000 mg), I (400 mg), Fe (1,200 mg), Mn (18,000 mg), Se (60 mg), and Zn (14,000 mg).

The chemical profile of the palm fronds used in this study is presented in Table 2. Following a 10-day adaptation period to the pelleted feed and standard veterinary prophylaxis (covering endo- and ectoparasites, PPR, and septicemia). Water and feed were provided ad libitum, with adequate ventilation and hygiene maintained throughout the 56-day experiment.

**Table 2:** Chemical profile of the palm fronds used in the experimental diets.

Nutrients	% of DM
DM	94.10
OM	86.26
ME, kcal/kg	2,280
CP	3.50
Starch	2.90
Ash	7.84
EE	3.70
NDF	53.90
ADF	33.10
Lignin	4.90
NFC	31.10
Ca	1.14
P	0.04

The nutrient profile was analyzed in duplicate.

### Preparation of palm fronds

Palm fronds (*Phoenix dactylifera L.*) were collected from local farms in Riyadh, Saudi Arabia. After collection, the fronds were manually cleaned to remove physical impurities and air-dried in the shade until a constant moisture content was achieved. The dried material was then mechanically ground using a mixer mill (Retsch GmbH, Haan, Germany). To ensure dietary homogeneity and uniform particle size, the ground material was sieved through a 1 mm mesh screen using a vibratory sieve shaker (Model 1868, Bertel, Caieiras, Brazil) before incorporation into the pelleted complete diets.

### Growth performance indices

Each experimental unit (two lambs per replicate pen) was weighed as an average to record initial BW at the initial (1 d), intermediate (28 d), and final (56 d) time points before the morning feeding using a floor electric scale (Hangzhou Co., Ltd., Zhejiang, China). Body weight gain (BWG = final minus initial BW) and average daily gain (ADG = final minus initial BW divided by trial period in days) were calculated for each pen during phase I (1–28 d), phase II (29–56 d), and the cumulative period (1–56 d) according to the method described by Keno et al. (20). During these same phases, daily feed intake (DFI = total diet offered minus refused diet divided by trial period in days) was estimated for each pen (21). These data were used to determine the feed conversion ratio (FCR), defined as DFI divided by ADG per experimental unit, following the methodology of Ellison et al. (22).

### Apparent nutrient digestibility

Following the initial 56-day growth performance trial, a nutrient digestibility study was conducted over an additional 14-day period, bringing the total experimental duration to 70 days. Eight lambs per group (n = 8; one lamb per replicate pen) were randomly selected and transferred to individual metabolic cages (1.2 m height × 0.6 m width) designed for the quantitative separation of feces and urine. The total collection method was employed, consisting of a 7-day adaptation period followed by a 7-day collection period. During the collection phase, DFI was recorded and total fecal output was collected, weighed, and a representative aliquot (10%) was sampled and stored at −20 °C for subsequent analysis. Chemical analyses of palm fronds, diets, and fecal samples were performed in triplicate according to AOAC (23) standards. Dry matter (DM) was determined by oven-drying at 105 °C, crude protein (CP) by the Kjeldahl method (N × 6.25), and ether extract (fat) by the Soxhlet method. Ash content was determined by incineration in a muffle furnace at 550–600 °C for 5 h. Fiber fractions, including neutral detergent fiber (NDF) and acid detergent fiber (ADF), were analyzed using an ANKOM Technology fiber analyzer (Macedon, NY, USA) as described by Van Soest et al. (24). Metabolizable energy of diets and palm fronds was calculated from gross energy values by applying a correction factor of 0.82 to the digestible energy, as proposed by Neto et al. (25). Calcium and phosphorus contents of diets and palm fronds were measured using atomic absorption spectrometry (26). Nutrient digestibility was calculated as [(nutrient consumed minus nutrient excreted) divided by total nutrient intake] multiplied by 100, using the previously described formula (27).

### Slaughter procedures and sampling

At the end of the 56-day feeding trial, eight remaining lambs per group (one lamb per replicate pen) were selected and weighed for slaughter to estimate intraruminal fermentation profiles and rumen histomorphology, as well as to evaluate carcass traits and meat quality. The lambs were fasted for 12 h with free access to water before being humanely slaughtered at a municipal abattoir.

### Intraruminal fermentation profiles

A portable pH meter (Sartorius PT-10, Germany) was used to record the pH in duplicate at the time of collection. To preserve fermentative metabolites, a 45 mL aliquot of strained ruminal fluid was transferred, after centrifugation at 14,000 × g for 10 min at 5 °C, into a 50 mL sterile polypropylene conical tube (Fair Lawn, NJ, USA). To stabilize the samples for subsequent ammonia nitrogen (NH3-N) and volatile fatty acid analysis, 5 mL of 0.2 N HCl was added to each tube to lower the pH and halt microbial activity. The tubes were then tightly sealed and stored at −80 °C until analysis. NH3-N concentrations were quantified to assess nitrogen availability and protein degradation using the phenol-hypochlorite colorimetric method (28). Analysis was performed on a spectrophotometer (PerkinElmer, MA, USA) after acidification with 0.1 N HCl. The volatile fatty acid profile, specifically the concentrations of acetic, propionic, and butyric acids, was evaluated (11). For sample preparation, 5 mL of the supernatant was stabilized with 1 mL of 25% metaphosphoric acid and centrifuged again (14,000 × g at 5 °C). The resulting 1 mL filtered supernatant was transferred to Agilent chromatography vials for analysis by gas chromatography–mass spectrometry (Agilent Technologies, Series 1,260).

### Rumen histomorphology assessment

A 2-cm^2^ tissue segment was excised from the ventral sac of the rumen and rinsed in physiological saline. The specimens were stabilized in 10% neutral buffered formalin for 72 h. Systematic dehydration and paraffin embedding were performed using an automated tissue processor (Tissue-Tek VIP 5 Jr., Sakura, Japan). Sections 5 μm thick were then prepared using a precision microtome (Leica Biosystems, Germany). After hematoxylin and eosin staining, an optical microscope with an image analysis system (Leica Imaging Systems Ltd., Cambridge, UK) was used to quantify structural parameters. Measured variables included the height, width, and density of the papillae (29). Absorptive capacity was estimated by calculating individual and total papilla surface areas based on the geometric measurements described by Sohail et al. (30).

### Carcass and meat characteristics evaluation

Hot carcass weight was measured immediately after removing non-carcass components. Non-carcass components and various internal organs were weighed individually to calculate the non-carcass components to hot carcass ratio (NCC/HC). Carcasses were chilled at 5 °C for 24 h to obtain the cold carcass weight. The carcass compactness index (CCI, kg/cm = cold carcass weight divided by internal carcass length) was calculated (31). Cold shrink was calculated as the difference between hot and cold carcass weights, divided by hot carcass weight, then multiplied by 100 (32). Dressing percentage was calculated as hot carcass weight divided by slaughter weight multiplied by 100 (32). Fat depots, including pericardial, kidney knob fat, and omental fat, were weighed and expressed as a percentage of slaughter weight. Backfat and body wall fat were also collected. The right half of each carcass was divided into primary wholesale cuts (shoulder, rack, loin, leg, foreshank and breast). Each section was weighed and reported as a percentage of the side weight (33).

Meat quality parameters were assessed on the longissimus dorsi muscle. Water-holding capacity was determined using the filter paper press method and expressed as a percentage, following the method described by Awawdeh et al. (31). Cooking loss was measured by boiling samples on an electric grill (Princess, 2,321, Netherlands) until an internal temperature of 75 °C was reached and expressed as a percentage (cooking loss = the difference between the initial and cooked weight, divided by the initial weight, multiplied by 100), according to the method of Mahgoub et al. (33). The Myofibril Fragmentation Index was determined spectrophotometrically at 540 nm after homogenization in a chilled buffer (34). Shear force and texture profile analysis (hardness, springiness, cohesiveness, and chewiness) were quantified using a TA-HD Texture Analyzer (Stable Micro Systems Ltd., UK) fitted with a Warner-Bratzler blade. Cooked muscle samples (2 cm^2^) were compressed to 50% of their original height. Results were analyzed according to previously established textural protocols (34).

### Economic evaluation

To assess the financial viability of incorporating palm fronds into lamb diets, a comprehensive economic analysis was conducted. Key indicators, including feed expenditure, production cost per kilogram of meat, gross revenue, and net profit, were measured. Advanced metrics such as relative economic efficiency, return on investment, profit margin, and price safety threshold were also determined using established econometric methods (26, 35).

Gross revenue was calculated based on carcass weight and the prevailing market price per kilogram. Net profit was defined as the difference between total revenue and total production costs. Relative economic efficiency was assessed by comparing the economic efficiency of each palm frond inclusion group (8, 16, and 24%) to the control group (0% palm frond), with efficiency defined as the ratio of net return to total expenditure. Profitability was further evaluated using return on investment, calculated as the percentage of net profit relative to total costs. The profit rate was expressed as the proportion of net profit within total revenue. The price safety limit, representing the maximum allowable feed cost per kilogram before incurring a loss, was estimated by dividing total revenue (minus non-feed costs) by total feed consumption per animal.

### Statistical analysis

Growth performance data were analyzed using the replicate pen as the experimental unit (*n* = 8 per group). In contrast, measurements of nutrient digestibility, ruminal fermentation, rumen histomorphology, and carcass quality were analyzed using the individual lamb as the experimental unit (*n* = 8 per group). To ensure the study was sufficiently powered, a *post hoc* power analysis was conducted using G*Power 3.1. Based on the observed Cohen’s d effect sizes (*n* = 16 lambs per group), the achieved power to detect significant differences at *α* = 0.05 ranged from 0.72 to 0.91, confirming that the sample size was adequate for the parameters measured. Before analysis, all datasets were tested with the Shapiro–Wilk and Levene’s tests to verify the assumptions of normality and homogeneity of variance, respectively.

All data were analyzed by one-way analysis of variance using the general linear model procedure of SAS Institute Inc., Cary, NC (36) according to the following statistical model:

Yij=μ+Gi+eij

Where: Y*ij* is the observed value; *μ* is the overall mean; G*i* is the fixed effect of palm frond inclusion level (i = 0, 8, 16, and 24%); e*ij* is the random residual error.

Significant differences between group means were determined using Tukey’s test, with statistical significance set at *p* < 0.05. All results are presented as mean values with the standard error of the mean (±SEM).

## Results

### Growth performance indices

Growth performance indices of Awassi lambs fed varying levels of palm frond in pelleted complete diets are shown in Table 3. At the intermediate (28 d) and final (56 d) phases, BW was highest in the 16% palm frond group (*p* = 0.022 and *p* = 0.007; respectively) compared to the 0 and 24% palm frond groups. This trend was also observed in cumulative BWG and ADG (1–56 d), where group C (16% palm frond) had higher values (*p* = 0.018) than the 0 and 24% palm frond groups.

**Table 3:** Growth performance indices of Awassi lambs fed varying levels of palm frond in pelleted complete diets.

**Parameter** ^**1**^	**Palm fronds level** ^**2**^				**SEM** ^**3**^	***p*-value**
	A (0%)	B (8%)	C (16%)	D (24%)		
BW (kg)						
Initial (1 d)	31.67	31.71	31.81	31.59	0.102	0.523
Intermediate (28 d)	41.10^b^	41.71^ab^	42.69^a^	41.17^b^	0.309	0.022
Final (56 d)	50.53^b^	51.50^ab^	52.17^a^	50.46^b^	0.283	0.007
BWG (kg)						
Phase I (1–28 d)	9.43	10.00	10.88	9.58	0.341	0.063
Phase II (29–56 d)	9.43	9.79	9.48	9.29	0.381	0.824
Cumulative Period (1–56 d)	18.87^b^	19.79^ab^	20.36^a^	18.87^b^	0.298	0.018
ADG (g/d)						
Phase I (1–28 d)	336.90	357.14	388.49	342.06	12.20	0.064
Phase II (29–56 d)	336.90	349.60	338.49	331.75	13.65	0.822
Cumulative Period (1–56 d)	336.90^b^	353.37^ab^	363.49^a^	336.90^b^	5.300	0.018
DFI (kg/d)						
Phase I (1–28 d)	1.74^a^	1.47^b^	1.39^b^	1.52^ab^	0.052	0.008
Phase II (29–56 d)	1.84	1.75	1.76	1.79	0.068	0.818
Cumulative Period (1–56 d)	1.79^a^	1.61^b^	1.58^b^	1.65^ab^	0.038	0.022
FCR (g/g)						
Phase I (1–28 d)	5.16^a^	4.17^ab^	3.59^b^	4.45^ab^	0.254	0.014
Phase II (29–56 d)	5.44	5.03	5.21	5.39	0.203	0.506
Cumulative Period (1–56 d)	5.30^a^	4.55^bc^	4.34^c^	4.91^ab^	0.117	0.002

Means within a row possessing divergent superscript letters (a–b) differ significantly (*P* < 0.05) according to Tukey’s test analysis. ^1^BW = body weight, BWG = body weight gain, ADG = average daily gain, DFI = daily feed intake, and FCR = feed conversion ratio. ^2^A = pelleted complete diet with 0% inclusion of palm fronds; B = pelleted complete diet with 8% inclusion of palm fronds; C = pelleted complete diet with 16% inclusion of palm fronds, and D = pelleted complete diet with 24% inclusion of palm fronds. ^3^SEM: Pooled Standard Error of the Mean (*n* = 8 pens per level).

Lambs fed the 8 and 16% palm frond diets had lower DFI than the 0% palm frond group (group A) during phase I (*p* = 0.008) and the cumulative period (p = 0.022). Consequently, the FCR was significantly improved (*p* = 0.002) in lambs fed a 16% palm frond level throughout the entire 56-day duration compared to the 0% palm frond group.

### Apparent nutrient digestibility

Nutrient digestibility of Awassi lambs fed varying levels of palm frond in pelleted complete diets is shown in Table 4. The DM digestibility in group C (16% palm frond) was higher (*p* = 0.021) than in the 0 and 8% palm frond groups. Lambs fed the 8 and 16% palm frond diets had higher (*p* = 0.038) CP digestibility compared to the 0 and 24% palm frond groups. NDF and ADF digestibility in the 16 and 24% palm frond groups was higher (*p* = 0.034 and *p* = 0.039; respectively) than in the 0 and 8% palm frond groups. Additionally, there was no significant effect (*p* > 0.05) on ash and fat digestibility among palm frond levels (groups A-D).

**Table 4:** Nutrient digestibility of Awassi lambs fed varying levels of palm frond in pelleted complete diets.

Parameter (%)^1^	Palm fronds level^2^				SEM^3^	*p*-value
	A (0%)	B (8%)	C (16%)	D (24%)		
DM	77.19^b^	78.80^b^	83.51^a^	80.77^ab^	1.250	0.021
CP	79.99^b^	85.08^a^	85.74^a^	79.79^b^	1.620	0.038
NDF	52.94^b^	56.49^b^	67.95^a^	66.12^a^	3.640	0.034
ADF	50.43^c^	54.77^c^	66.48^a^	60.10^b^	3.550	0.039
Ash	70.17	71.85	72.77	72.37	2.360	0.870
Fat	78.32	81.10	86.62	86.46	2.350	0.070

Means within a row possessing divergent superscript letters (a–b) differ significantly (*P* < 0.05) according to Tukey’s test analysis. ^1^DM = dry matter, CP = crude protein, NDF = neutral detergent fiber, and ADF = acid detergent fiber. ^2^A = pelleted complete diet with 0% inclusion of palm fronds; B = pelleted complete diet with 8% inclusion of palm fronds; C = pelleted complete diet with 16% inclusion of palm fronds, and D = pelleted complete diet with 24% inclusion of palm fronds. ^3^SEM: Pooled Standard Error of the Mean (*n* = 8 lambs per level).

### Fermentation and histomorphology of rumen

Intraruminal fermentation profiles and rumen histomorphology of Awassi lambs fed varying levels of palm frond in pelleted complete diets are shown in Table 5. There was no significant effect (p > 0.05) on pH value or NH3-N concentration in the rumens of lambs fed different palm frond levels (groups A–D). The concentrations of acetic acid, propionic acid, and total volatile fatty acids in group C (16% palm frond) were higher (*p* < 0.0001) than in the other palm frond levels. Additionally, the butyric acid concentration in group C (16% palm frond) was higher (p < 0.0001) than in the 0 and 8% palm frond groups. The acetic to propionic acid ratio (A: P) was higher (*p* = 0.047) in lambs fed 16 and 24% palm frond than in the 0% palm frond group.

**Table 5:** Intraruminal fermentation profiles and rumen histomorphology of Awassi lambs fed varying levels of palm frond in pelleted complete diets.

Parameter^1^	Palm fronds level^2^				SEM^3^	*P*-value
	A (0%)	B (8%)	C (16%)	D (24%)		
Rumen PH	6.58	6.56	6.61	6.45	0.123	0.815
NH3-N (mg/dl)	16.09	15.92	15.80	15.77	0.77	0.712
Volatile fatty acids						
Acetic acid (mM)	20.96^b^	21.85^b^	23.78^a^	21.65^b^	0.117	<0.0001
Propionic acid (mM)	11.50^b^	12.03^b^	12.76^a^	11.71^b^	0.145	<0.0001
Butyric acid (mM)	10.64^c^	11.34^bc^	12.93^a^	12.25^ab^	0.227	<0.0001
Total volatile fatty acids (mM)	43.10^b^	45.21^b^	49.47^a^	45.62^b^	0.272	<0.0001
Acetic to propionic acid ratio (A: P)	1.79^b^	1.82^ab^	1.86^a^	1.85^a^	0.018	0.047
Rumen histomorphology						
Papilla height (mm)	1.80^b^	1.73^b^	2.16^a^	1.94^ab^	0.091	0.011
Papilla width (mm)	0.29	0.31	0.35	0.33	0.015	0.088
Papilla surface area (mm^2^)	1.65^b^	1.67^b^	2.35^a^	2.02^ab^	0.126	0.0008
Density (n/cm^2^)	62.20^b^	78.60^a^	63.00^b^	60.58^b^	3.606	0.002
TSP (mm^2^/cm^2^)	65.47^b^	84.51^ab^	94.17^a^	77.18^ab^	6.520	0.048

Means within a row possessing divergent superscript letters (a–b) differ significantly (*P* < 0.05) according to Tukey’s test analysis. ^1^NH3-N = Ammonia-nitrogen and TSP = Total surface of papillae. ^2^A = pelleted complete diet with 0% inclusion of palm fronds; B = pelleted complete diet with 8% inclusion of palm fronds; C = pelleted complete diet with 16% inclusion of palm fronds, and D = pelleted complete diet with 24% inclusion of palm fronds. ^3^SEM: Pooled Standard Error of the Mean (*n* = 8 lambs per level).

Rumen histomorphology measurements, including papilla length and surface area, were higher (*p* = 0.011 and *p* = 0.0008; respectively) in lambs fed 16% palm frond compared to the 0 and 8% palm frond groups. There was no significant effect (*p* > 0.05) on papilla width among palm frond levels (groups A-D). Papilla density was higher (*p* = 0.002) in lambs fed 8% palm frond than at other palm frond levels. However, lambs fed 16% palm frond had higher TSP (*p* = 0.048) than the 0% palm frond group.

### Carcass characteristics and slaughter yields

Carcass characteristics of Awassi lambs fed different levels of palm frond in pelleted complete diets are shown in Table 6. Slaughter weight, hot carcass weight, and cold carcass weight in group C (16% palm frond) were higher (*p* < 0.05) than in the 0 and 24% palm frond groups. There was no significant effect (*p* > 0.05) on CCI and NCC/HC in lambs fed varying palm frond levels (groups A–D). Relative cold shrink was lower in lambs fed 16% palm frond, followed by those fed 8% palm frond, compared to the other groups (*p* < 0.0001). Dressing percentage was higher (*p* = 0.003) in lambs fed 16% palm frond than in the 0 and 24% palm frond groups, but did not differ significantly between the 8 and 16% or the 0 and 24% palm frond groups.

**Table 6:** Carcass characteristics of Awassi lambs fed varying levels of palm frond in pelleted complete diets.

Parameter^1^	Palm fronds level^2^				SEM^3^	*p*-value
	A (0%)	B (8%)	C (16%)	D (24%)		
Slaughter weight (kg)	50.38^b^	51.76^ab^	52.72^a^	50.90^b^	0.434	0.008
Hot carcass weight (kg)	26.10^b^	26.90^ab^	27.50^a^	26.16^b^	0.349	0.029
Cold carcass weight (kg)	25.50^b^	26.50^ab^	27.20^a^	25.70^b^	0.303	0.005
CCI (kg/cm)	0.40	0.41	0.38	0.40	0.009	0.056
NCC/HC	0.75	0.74	0.79	0.80	0.026	0.284
Cold shrink (%)	1.89^a^	1.54^b^	1.24^c^	1.60^a^	0.062	<0.0001
Dressing (%)	51.73^bc^	51.98^ab^	52.24^a^	51.39^c^	0.107	0.003

Means within a row possessing divergent superscript letters (a–b) differ significantly (*P* < 0.05) according to Tukey’s test analysis. ^1^CCI = Carcass compactness index in (kg/cm); NCC/HC = Non-carcass components to hot carcass ratio. ^2^A = pelleted complete diet with 0% inclusion of palm fronds; B = pelleted complete diet with 8% inclusion of palm fronds; C = pelleted complete diet with 16% inclusion of palm fronds, and D = pelleted complete diet with 24% inclusion of palm fronds. ^3^SEM: Pooled Standard Error of the Mean (*n* = 8 lambs per level).

### Wholesale cutting and internal fat depots

Carcass cutting and fat deposition of Awassi lambs fed varying levels of palm frond in pelleted complete diets are shown in Table 7. The relative weight of the shoulder was higher (*p* = 0.005) in lambs fed 16% palm frond than in the 0 and 24% palm frond groups. In contrast, the relative weights of other carcass cuts, including rack, loin, leg, foreshank, and breast, were not significantly affected (*p* > 0.05) by varying palm frond levels (groups A–D).

**Table 7:** Carcass cutting and fat deposition of Awassi lambs fed varying levels of palm frond in pelleted complete diets.

Parameter	Palm fronds level^1^				SEM^2^	*p*-value
	A (0%)	B (8%)	C (16%)	D (24%)		
Carcass cutting						
Shoulder (%)	25.70^c^	27.98^ab^	28.58^a^	26.79^bc^	0.395	0.005
Rack (%)	9.26	9.18	9.36	8.66	0.292	0.073
Loin (%)	14.54	14.14	14.16	14.67	0.397	0.747
Leg (%)	33.60	31.77	31.10	32.57	0.662	0.144
Foreshank and breast (%)	16.90	16.93	16.80	17.31	0.572	0.763
Fat deposition						
Backfat (mm)	3.79^b^	4.19^a^	4.09^ab^	3.34^c^	0.092	0.001
Body wall fat (mm)	5.84^b^	6.38^ab^	5.85^b^	7.13^a^	0.227	0.002
Pericardial fat %	0.18^a^	0.15^ab^	0.10^c^	0.11^bc^	0.010	0.001
Kidney knob fat %	1.01^a^	0.57^b^	0.41^b^	0.56^b^	0.068	0.001
Omental fat %	1.24	1.22	0.92	0.96	0.114	0.137

Means within a row possessing divergent superscript letters (a–b) differ significantly (*P* < 0.05) according to Tukey’s test analysis. ^1^A = pelleted complete diet with 0% inclusion of palm fronds; B = pelleted complete diet with 8% inclusion of palm fronds; C = pelleted complete diet with 16% inclusion of palm fronds, and D = pelleted complete diet with 24% inclusion of palm fronds. ^2^SEM: Pooled Standard Error of the Mean (*n* = 8 lambs per level).

For fat deposition, backfat increased in the 8% palm frond group and decreased in the 24% palm frond group compared to the 0% palm frond group (*p* = 0.001). Additionally, body wall fat was higher (*p* = 0.002) in lambs fed 24% palm frond than in the 0 and 16% palm frond groups. The relative weight of pericardial fat was lower (*p* = 0.001) in lambs fed 16% palm frond than in the 0 and 8% palm frond groups. Lambs fed varying palm frond levels (groups B–D) had lower (*p* = 0.001) kidney knob fat compared to the 0% palm frond group (group A). Relative weight of omental fat was not significantly affected (*p* > 0.05) in lambs fed varying palm frond levels (groups A–D).

### Physicochemical and textural properties of meat

Meat characteristics of Awassi lambs fed different levels of palm frond in pelleted complete diets are shown in Table 8. Cooking loss percentages were not significantly affected (*p* > 0.05) by varying palm frond levels (groups A–D). Water-holding capacity was lower (*p* = 0.003) in lambs fed 16% palm frond compared to the other palm frond levels. In contrast, lambs fed 24% palm frond had a lower (*p* = 0.0002) myofibril fragmentation index than the 0 and 8% palm frond groups. Shear force increased in the 16% palm frond group and decreased in the 24% palm frond group compared to the 0 and 8% palm frond groups (*p* < 0.0001). Texture profile analysis parameters, including hardness, cohesiveness, and chewiness, were not significantly affected (p > 0.05) by varying palm frond levels (groups A–D). However, springiness was higher (*p* = 0.026) in lambs fed 16% palm frond than in the 0% palm frond group.

**Table 8:** Meat characteristics of Awassi lambs fed varying levels of palm frond in pelleted complete diets.

Parameter	Palm fronds level^1^				SEM^2^	*p*-value
	A (0%)	B (8%)	C (16%)	D (24%)		
Cooking loss (%)	39.85	37.60	36.84	38.12	0.906	0.158
Water-holding capacity (%)	36.25^a^	35.70^a^	34.48^b^	35.88^a^	0.293	0.003
Myofibril fragmentation index	79.88^a^	78.92^ab^	73.28^bc^	68.16^c^	1.568	0.0002
Shear force (N/cm^2^)	5.96^b^	6.07^b^	7.53^a^	4.43^c^	0.257	<0.0001
Texture profile analysis						
Hardness (N)	1.69	1.90	1.91	1.75	0.275	0.914
Springiness	0.70^b^	0.83^ab^	1.07^a^	0.96^ab^	0.081	0.026
Cohesiveness	0.43	0.45	0.57	0.66	0.075	0.163
Chewiness	0.93	0.55	1.05	1.29	0.232	0.196

Means within a row possessing divergent superscript letters (a–b) differ significantly (*P* < 0.05) according to Tukey’s test analysis. ^1^A = pelleted complete diet with 0% inclusion of palm fronds; B = pelleted complete diet with 8% inclusion of palm fronds; C = pelleted complete diet with 16% inclusion of palm fronds, and D = pelleted complete diet with 24% inclusion of palm fronds. ^2^SEM: Pooled Standard Error of the Mean (*n* = 8 lambs per level).

### Economic profitability and efficiency analysis

Economic evaluations of Awassi lambs fed different levels of palm frond in pelleted complete diets are shown in Table 9. The inclusion of palm frond significantly affected the financial dynamics of the production cycle, mainly by reducing input costs and increasing profit margins. The total feed cost per animal decreased linearly as palm frond inclusion increased, with group D (24%) showing the lowest expenditure (30.36 USD) compared to the control group (38.66 USD). Although feed costs were lower, the net return per animal was highest in Group C (16% inclusion), reaching 265.48 USD, which was significantly higher than the 217.12 USD recorded for group A. Efficiency indices further highlighted the benefits of the 16% inclusion level. The return on investment peaked in group C (81.89%), followed by group B (71.55%) and group D (69.77%), all exceeding the 65.40% observed in the control group. Similarly, the total operating ratio and equilibrium quantity were lowest in Group C (0.55 and 0.50, respectively), indicating highly efficient resource conversion into revenue.

**Table 9:** Economic evaluation of Awassi lambs fed varying levels of palm frond in pelleted complete diets.

Parameter	Units	Palm fronds level^1^			
		A (0%)	B (8%)	C (16%)	D (24%)
Cost per kilogram of feed	*USD	0.36	0.34	0.33	0.31
Average final BW	kg	50.53	51.50	52.17	50.46
Average daily consumption (days 1–56)	kg	1.79	1.61	1.58	1.65
Price of 3-month-old lambs	USD	266.67	266.67	266.67	266.67
Care costs	USD	26.67	26.67	26.67	26.67
Total feed cost per animal	USD	38.66	33.23	30.84	30.36
Total costs per animal	USD	332.00	326.56	324.17	323.69
Average carcass weight	kg	25.74	26.26	27.64	25.76
Selling price per kilogram	USD	21.33	21.33	21.33	21.33
Total return per animal	USD	549.12	560.21	589.65	549.55
Net return per animal	USD	217.12	233.65	265.48	225.85
Economic efficiency	-	0.65	0.72	0.82	0.70
Relative economic efficiency	%	100.00	109.40	125.22	106.69
Return on investment	%	65.40	71.55	81.89	69.77
Total operating ratio	%	0.60	0.58	0.55	0.59
Equilibrium quantity	-	0.55	0.53	0.50	0.54
Equilibrium point	USD	302.68	298.59	295.49	297.54
Price safety threshold	%	44.88	46.70	49.89	45.86
Rate of profit	%	39.54	41.71	45.02	41.10

^*^The prices are given for the year 2025 AD. Palm fronds price per kg, 0.06 USD. ^1^A = pelleted complete diet with 0% inclusion of palm fronds; B = pelleted complete diet with 8% inclusion of palm fronds; C = pelleted complete diet with 16% inclusion of palm fronds, and D = pelleted complete diet with 24% inclusion of palm fronds.

The relative economic efficiency peaked in group C at 125.0% compared to the control group, followed by group B at 109.40% and Group D at 106.69%. This indicates that all inclusion levels improved efficiency over the baseline. Additionally, the price safety threshold was highest in the 16% inclusion group at 49.89%, suggesting that this dietary formulation offers the greatest resilience against market fluctuations in feed prices compared to the control group at 44.88%. As a result, the profit rate was highest in group C at 45.02%, confirming that 16% palm frond inclusion is the most economically viable strategy among the evaluated dietary treatments.

## Discussion

The use of date palm fronds as a sustainable roughage source in ruminant nutrition is gaining attention due to the environmental and economic needs of arid regions (37). Our study found that replacing conventional fiber sources, specifically wheat straw and alfalfa hay with palm fronds in pelleted complete diets significantly improves the biological and economic efficiency of Awassi lambs. The results indicate that growth performance, including BW, BWG, and ADG, was higher in lambs fed a 16% palm frond level throughout the 56-day period. The significant increase in ADG observed at the 16% palm frond inclusion level can be attributed to an optimal balance between dietary effective fiber and fermentable energy. Soliman et al. (13) reported that pelleted complete diets containing palm frond can provide greater average daily gain than traditional roughage. The reduced performance at the 24% level compared to the 16% level of palm frond may be due to the bulk effect of high lignocellulosic materials, which can increase ruminal fill and limit net energy intake (38). This aligns with Mahgoub et al. (33), who reported that while small ruminants are resilient to low-quality roughage, excessive fiber inclusion in total mixed rations can dilute metabolizable energy concentration, thereby limiting weight gain.

Notably, DM intake was lower in the 8 and 16% palm frond groups, yet these groups maintained or exceeded the growth of the control group. This resulted in a significantly improved FCR, especially at the 16% palm frond level. However, these findings may suggest that the pelleting process effectively mitigated the low palatability and low bulk density typically associated with palm fronds, facilitating better nutrient utilization. Pelleting reduces particle size, increases the surface area for microbial attachment, and enhancing the passage rate of fibrous particles (39). The higher nutritional density achieved through pelleting allows lambs to meet their physiological requirements with a lower volume of feed (40). The formulation of the 16% diet appears to have achieved superior synchronization between fermentable energy and nitrogen available to rumen microbes, a key factor in improving FCR in growing lambs (41).

The digestibility data suggests that palm fronds may contribute to the metabolic efficiency of the animal. The DM digestibility indicates that a 16% level of palm frond may provide the ideal balance between structural roughage and fermentable energy. In ruminant nutrition, the digestibility of DM is often limited by the slowest-digesting component, which is usually the fiber source (42). In addition, fiber fractions (NDF and ADF) were higher at the 16 and 24% palm frond levels than the basal diet (0% palm frond). These may suggest that the lambs’ rumen microbiota adapted efficiently to the lignocellulosic nature of the palm frond. Higher digestibility fiber fractions were supported by the highly total volatile fatty acids observed in lambs fed 16% palm fronds. The physical processing of palm fronds into a 200 μm particle size, followed by pelleting, successfully bypassed the traditional limitations of palm-based roughage. This aligns with findings by Kholif et al. (37), who reported that reducing the particle size of date palm leaves enhances the surface area for microbial colonization, thereby accelerating the rate of fermentation. The CP digestibility was higher in the 8 and 16% palm frond levels compared to other diets. Date palm frond are known to contain secondary metabolites, including tannins and phenolic compounds (43). While high levels of tannins can impair protein digestion, low to moderate concentrations can exert a “protein-shielding” effect, protecting dietary protein from excessive ruminal degradation and increasing the flow of non-ammonia nitrogen to the abomasum (44).

The lack of significant variation in ruminal pH across treatments indicates that palm fronds provide an effective buffering capacity, which likely may be due to the stimulation of rumination and salivary flow despite the small particle size (200 μm). Maintaining a pH between 6.45 and 6.61 is vital for the stability of cellulolytic bacteria, which are sensitive to acidic environments (45). Similarly, the consistent NH3-N concentrations suggest that the nitrogen release from the pelleted diet was well-synchronized with the energy available for microbial protein synthesis. This homeostasis is consistent with the findings of Mahgoub et al. (33), who observed that palm frond do not compromise the rumen’s buffering integrity when incorporated into pelleted complete diets.

Volatile fatty acids provide up to 50–85% of the metabolic energy for ruminants (15, 46). The current study showed that concentrations of acetic, propionic, and total volatile fatty acids in the 16% palm frond indicate a more robust fermentation profile. The increased butyric acids and acetic-to-propionic acid ratio in the lambs fed may suggest a shift in fermentation that supports both energy production and fiber digestion efficiency. These volatile fatty acids play a multifaceted role in regulating ruminal physiological functions and participate directly in the morphological remodeling of the rumen epithelium by promoting the proliferation and renewal of epithelial cells (17). However, our results show a higher papilla length and surface area in a 16% palm frond level within the complete diet. This may be due to a direct physiological effect of the elevated volatile fatty acid concentrations, particularly butyrate and propionate, which are known to act as chemical stimulants for the growth of the rumen epithelium (47). The increase in total surface area of papillae at the 16% palm frond level indicates a heightened absorptive capacity. By increasing the surface area, the lambs are better equipped to absorb the higher volume of volatile fatty acids produced, thereby preventing accumulation that could lead to sub-acute ruminal acidosis and ensuring a steady flow of energy to the host (48). The density of papillae was highest by 8% at the palm frond level, but surface area peaked by 16% at the palm frond level, suggesting that the rumen compensates for fewer papillae by increasing their individual size to maximize absorption—a phenomenon observed in high-performing lambs fed processed agricultural residues (49).

The significantly higher slaughter, hot, and cold carcass weights in the 16% palm frond level are direct reflections of the improved growth performance and nutrient digestibility discussed previously. In addition, the higher dressing percentage by 16% at the palm frond level compared to the 0 and 24% palm frond levels may suggest a more favorable ratio of carcass to non-carcass components. This is consistent with a study by dos Santos Cabral et al. (50) that showed carcass characteristics, including hot and cold carcass weights were increased in lambs fed palm frond as part of a complete diets. In contrast, palm fronds alone had no effect on carcass characteristics (6, 11). Furthermore, the lower relative cold shrink in the 16% followed by 8% palm frond indicates better moisture retention during the cooling process, which is a critical factor for retail meat yield and profitability (51). The uniformity in the relative weights of high-value cuts (rack, loin, leg) across all groups is a positive indicator, suggesting that palm frond inclusion does not negatively alter the muscle distribution of the Awassi lambs. The increase in the relative weight of the shoulder in 16% palm frond level may be attributed to the overall increase in slaughter weight and lean tissue accretion facilitated by the optimized energy-to-protein ratio. This consistency in carcass composition aligns with Jaborek et al. (52), who reported that alternative fibrous sources in pelleted diets tend to support standard carcass development as long as energy density is maintained.

The reduction in kidney knob fat for all palm frond levels is a favorable trait, as internal fats are generally considered waste in commercial processing. Conversely, the variation in backfat and body wall fat suggests that the 16% palm frond level balances subcutaneous fat cover (backfat) without excessive adiposity. The lower pericardial fat in 16% palm frond suggests a partitioning of energy toward muscular growth rather than visceral fat storage. These findings are consistent with the metabolic effects of volatile fatty acids, higher propionate levels primarily support gluconeogenesis and muscle development, whereas excessive dietary fiber (24% palm frond level) may shift the energy balance toward different lipid storage sites (53). The water-holding capacity was lower in 16% palm frond level, which often correlates with the rate of post-mortem pH decline and protein denaturing. Despite this, the lack of significant differences in cooking loss suggests that the culinary yield of the meat remains comparable across all diets. The meat from lambs in the 16% palm frond level had higher shear force and springiness. While shear force is an objective measure of tenderness, the values observed remain within the acceptable range for quality lamb meat. The increase in shear force at this level may be a secondary effect of the significantly higher average daily gain observed in these lambs. Rapid growth in small ruminants is often associated with larger muscle fiber diameters and a potential shift in collagen cross-linking density, which can objectively increase resistance to shearing (54). The lower myofibril fragmentation index in the 24% palm frond level suggests a reduction in proteolysis during aging at the highest fiber level. The stability of hardness, cohesiveness, and chewiness across all groups indicates that the inclusion of palm fronds does not adversely affect the “mouthfeel” or eating quality of the meat, which is essential for consumer acceptance (55).

From an economic perspective, lambs fed 24% palm frond had the lowest feed expenditure, but their net return per animal was lower than those fed 16% palm frond. This indicates that at the 16% inclusion level, lambs achieved a superior biological response, resulting in higher marketable weight and effectively offsetting the slightly higher cost compared to the 24% group. The peak return on investment and relative economic efficiency in group C demonstrate a significant competitive advantage over traditional feeding regimes. In economic terms, a return on investment improvement of nearly 16.5% over the control group suggests high capital efficiency. The total operating ratio of 0.55 in group C further supports this, indicating that for every dollar of revenue generated, only 55 cents are consumed by operating expenses. Such a ratio is highly favorable in commercial livestock operations, providing a larger buffer for fixed costs and net profit. These results are consistent with Ayoo and Bonti-Ankomah (56), who noted that lambs fed agriculture by-products can enhance the economic sustainability of farms by reducing dependency on imported grains. Furthermore, research indicates that the use of locally available date palm leaves as a roughage source is a viable and useful option for reducing feed costs while maintaining productivity (6). The price safety threshold for group C suggests that an operation using 16% palm fronds can withstand a nearly 50% surge in feed market prices before incurring losses.

## Conclusion

In conclusion, integrating date palm fronds into pelleted complete diets for lambs provides an effective and sustainable alternative to traditional roughage sources. An optimized inclusion level of 16% palm fronds enhances growth performance and feed utilization efficiency by increasing the ruminal absorptive surface area and stimulating volatile fatty acid production in Awassi lambs. This dietary strategy supports standard carcass development, promotes leaner growth, and reduces visceral fat accumulation. Additionally, including palm fronds at 16% significantly lowers input costs and maximizes net returns, offering the livestock sector greater resilience to global feed price volatility. However, utilizing palm fronds through precision pelleting presents a circular economy solution for the livestock sector in arid regions. Further research is needed to evaluate the long-term effects of this dietary strategy on meat oxidative stability and the molecular pathways of nutrient absorption in the rumen.

## Data Availability

The original contributions presented in the study are included in the article/supplementary material, further inquiries can be directed to the corresponding author/s.
